# Effect of Direct Powder Forging Process on the Mechanical Properties and Microstructural of Ti-6Al-4V ELI

**DOI:** 10.3390/ma14164499

**Published:** 2021-08-11

**Authors:** Sébastien Germain Careau, Bernard Tougas, Elena Ulate-Kolitsky

**Affiliations:** 1 Centre de Métallurgie du Québec, Trois-Rivières, QC G9A 5E1, Canada; bernard.tougas@cegeptr.qc.ca; 2 Département de Physique, Université du Québec à Trois-Rivières, Trois-Rivières, QC G8Z 4M3, Canada; elena@irh.ca

**Keywords:** direct powder forging, thermomechanical processing, Ti-6Al-4V, microstructure evaluation, mechanical properties

## Abstract

The study of powder metallurgy processing methods for titanium represents a promising avenue that can respond to a growing demand. This work reports the feasibility of direct powder forging (DPF) as a method to process large spherical Ti-6Al-4V powder into wrought products with noteworthy properties and physical characteristics. Direct powder forging is a thermomechanical process that imparts uniaxial loading to an enclosed and uncompacted powder to produce parts of various sizes and shapes. Stainless steel canisters were filled with prealloyed Ti-6Al-4V powder and consolidated through a multi-step open-die forging and rolling process into wrought DPF bars. After DPF, annealing was performed in the upper α+β phase. The results show that full consolidation was achieved and higher mechanical properties than the Ti-6Al-4V grade F-23 requirements in annealed conditions were obtained. The results also show that direct powder forging of spherical titanium powder could produce wrought mill products and exhibit some potential for further investigation for industrial applications.

## 1. Introduction

The increasing demand for new industrial applications of titanium can be attributed to its desirable properties such as high specific mechanical properties, high corrosion resistance and high biocompatibility [1]. In fact, titanium alloys are widely used by the aerospace, automotive, chemical and medical industries. Over the recent decades, many technologies have been developed to produce titanium parts by powder consolidation [2]. Hot isostatic pressing (HIP) has been one of the predominant powder metallurgy (PM) processes. In recent research, HIP was used to process large size Ti-6Al-4V powder (53–250 µm) from the retained particles after screening out the fine powder used in additive manufacturing (AM) [3]. The author reported that full density can be achieved and that the compacts presented a finer microstructure than those obtained with conventional casting techniques. Thus, the fine microstructure led to the enhancement of the tensile strength.

Although HIP is currently the most efficient route for full consolidation of titanium powder [2,4], other consolidation techniques have been development and are currently being improved. Direct powder forging (DPF) provides opportunities to address the limitation of conventional casting and PM processes. This technique was introduced in the 70’s by Olssom et al. [5] to consolidate tool steel powder into bar stock without the use of isostatic compaction. They reported that full density was obtained by forging and rolling the filled and sealed canister. In addition, the authors used oxygen getter (titanium) to prevent oxidation during thermomechanical processing. The authors concluded that bonding between particle cannot be achieved without the use of a proper oxygen getter. Over the years, improvement in technology has led to more efficient tools and equipment, resulting in a better control of processing parameters. More recently, researchers have worked on the feasibility of producing nickel superalloys with DPF from fine particles (30 μm) [6,7,8]. Results showed that full density can be achieved and that the microstructure was finer than that obtained by traditional HIPping process. In addition, they showed how DPF improved processing time and how the oxygen getter could be avoided. They also demonstrated an important advantage of DPF, it only requires conventional forging equipment. Therefore, DPF can lead to enhanced mechanical properties without the need for expensive equipment and offer a new perspective for industrial applications. Lastly, the authors pointed out the potential of DPF for near-net-shape parts. DPF samples can be forged into preformed parts with a close-die forging step.

There is still little information available about the DPF process and its influence on the properties of fully consolidated PM parts. Thus, further research is needed to provide a better description of DPF and its potential. The current study aims to extend the material knowledge of the DPF process by evaluating the feasibility of utilizing DPF to produce wrought titanium alloys and to characterize its influence on the final part. The resulting chemical composition, mechanical properties and microstructure are discussed in the present article.

## 2. Materials and Methods

### 2.1. Specimen Preparation

The DPF process was carried out using pre-alloyed Tekna Advance Material plasma atomized Ti-6Al-4V (Grade 23) powder. The spherical powder size distribution was between 90 µm and 250 µm. These large spheroidal particles came from offcuts of their common production. Its chemical composition is presented in Table 1.

**Figure 1:**
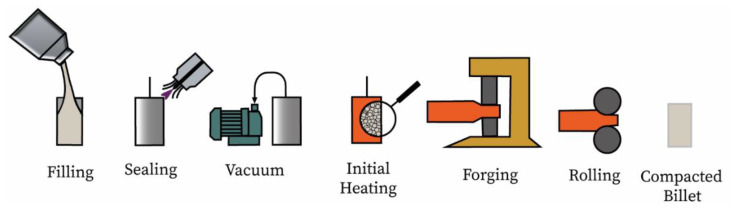
Presentation of the DPF sequence of operations used in the current study to achieve consolidation.

**Figure 2:**
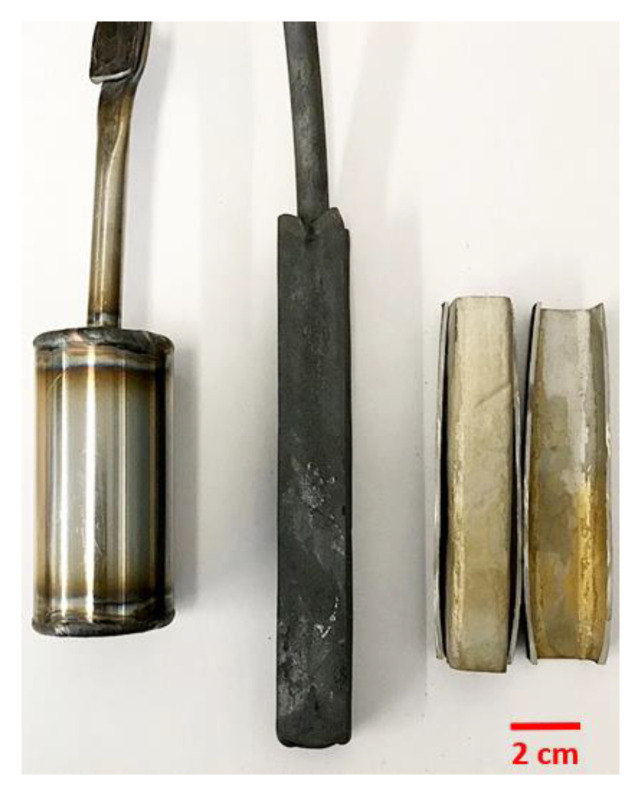
Photograph presenting a new sealed canister (**left**), a forged billet (**center**) and a cut-out sample (**right**).

The canisters used for the DPF were made from 316 L sanitary grade stainless steel (CFF Stainless Steels Inc., Montreal, QC, Canada). This stainless steel was selected because of its good weldability and excellent ductility at forming temperature. The canister’s dimensions were an outer diameter of 38.1 mm, a length of 101.6 mm and a wall thickness of 1.3 mm. These were cleaned with alcohol (ethanol) and filled with the powder. The canisters were vibrated to maximize powder capacity. After this step, the canisters were filled with argon to prevent oxidation of the powder or can during the welding. Next, the canisters were vacuumed to around 0.4 Pa and sealed by crimping the cap stem. The canisters were then placed at 1000 °C for 3 h prior to forging to ensure initial particles bonding. After this, multi-step thermomechanical processing was performed on the canister. The samples were heated up to 1100 °C and a 150 T open-die hydraulic press (CMQ, Trois-Rivières, Canada) and a 15 HP rolling mill (Stanat Manufacturing Company, Long Island City, NY, USA) were used to apply the deformation ensuring that the canister’s temperature never dropped below 900 °C. Total deformation of 50% was needed to consolidate the Ti-6Al-4V titanium alloy powder. A better overview of the whole DPF process is illustrated in Figure 1. Pictures of the canister at different stages of the DPF process are presented in Figure 2. After consolidation, annealing under argon was performed at 925 °C for 2 h. Samples were then left to cool down in the furnace.

### 2.2. Specimen Characterization

**Figure 3:**
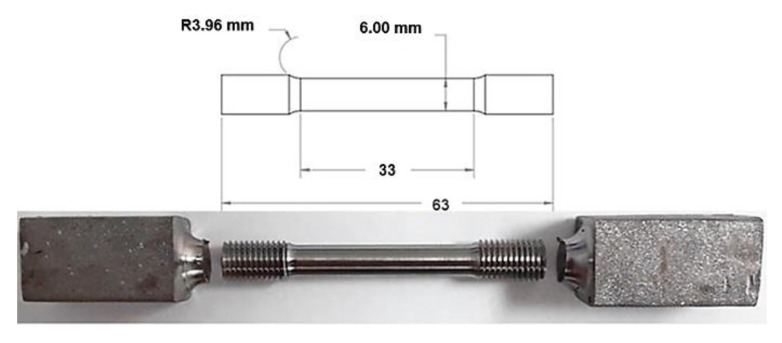
Presentation of the excised tensile specimen from the DPF bar.

The tensile properties were evaluated at a strain rate of 0.015 mm/min with a Tinius Olsen Super L machine (Tinius Olsen TMC, Horsham, PA, USA). Samples were prepared following the ASTM E8-16a standard [9]. As shown in Figure 3, thread ends rounded tensile bars were machined from the 16 × 16 × 150 mm forged and annealed bars. These ingots were the ones obtained from the small canister size.

The relative density before DPF was determined according to the MPIF industry standard 42, and the relative density after DPF was evaluated on 7 samples by the Archimedes principle according to the ASTM B962-17 [10]. Before measurement, all specimens were machined to remove any possible surface contamination from the canister. Microstructural observations were carried out on the cross-sections of the DPF bars. These were polished to a 0.05 µm surface finish and etched with Kroll regent. Observations were carried out with a Nikon Eclipse ME600 optical microscope (Nikon Instruments Inc., Melville, NY, USA) using CLEMEX Vision PE software (V8.1) and a Hitachi SU3500 scanning electron microscope (SEM, Hitachi High-Technologies Corporation, Tokyo, Japan) equipped with an Oxford Instruments S-Max energy dispersive spectrometer (EDS, Oxford Instruments NanoAnalysis, Concord, MA, USA) and AZtec software (V3.3). In accordance with ASTM E2371-13 [11], chemical composition was measured on the forged and annealed cross-sections with an optical emission spectrometer (OES) from Thermo Scientific (ARL 3460 Metals Analyzer, Thermo Fisher Scientific Inc, Mississauga, ON, Canada). The oxygen content was evaluated via inert gas fusion using a Bruker G8 Galileo (Bruker Elemental GmbH, Kalkar, Germany) conforming to ASTM E1409-13 [12]. The crystal structure was evaluated using a Bruker D8 X-ray diffractometer (XRD, Bruker Elemental GmbH, Kalkar, Germany) with a CuKα radiation source. Finally, phase proportion was carried out by Rietveld refinement method using TOPAS software (V6.0) and by image analysis using software ImageJ.

## 3. Results and Discussion

### 3.1. Physical Properties and Chemical Composition

**Figure 4:**
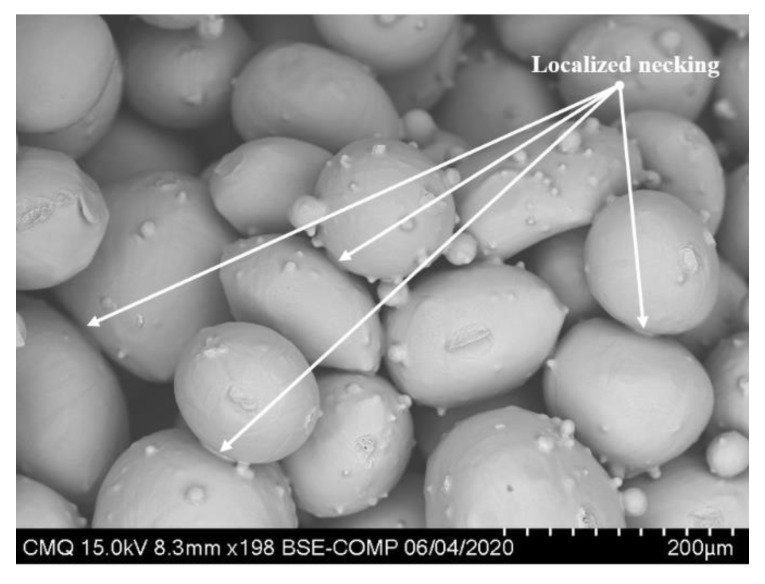
SEM micrographs of the Ti-6Al-4V spherical powder after the initial heating.

Figure 4 shows the powder after the initial heating step, i.e., powder was placed at 1000 °C for 3 h. The goal of this step was to form initial bounds between the particles and thus limit powder displacement within the canister in the first forging steps. As seen in the micrograph, the initial heating has promoted localized necking between particles. Neck formation is expected at the particles’ contact surfaces. Without compaction, the loose powder develops limited interparticle bonds. The initial heating step promoted the reduction in the total surface energy by increasing the dihedral angle at the contact point, which results in the formation of small interparticle bonding between particles [13]. Furthermore, since large particles have less surface area and a bigger radius, which corresponds to less surface energy and smaller driving forces for particles’ surface diffusion, neck growth was slow and, consequently, the neck sizes were limited at the end of this step.

The sample’s density before and after DPF is presented in Table 2. When compared to the theoretical value, we can conclude that full densification was obtained. Results are comparable to those obtained from conventional hot isostatic processing and are similar to the results reported by researchers for nickel superalloys DPF samples [6,8].

The oxygen content of the virgin powder and the DPF samples is shown in Table 3. Results show a negligible increase from the starting condition. This validates that the methodology proposed in this paper has the potential for critical application that require severe tolerance for interstitial such as grade 23 ELI (0.13 wt-% max). However, caution must be taken. In the work carried out by Peter et al. on direct powder rolling (DPR), similar steel canisters were employed to insert multi-layers of pre-compacted sheets of titanium for subsequent thermomechanical processing [14]. They reported that this route heavily increased the oxygen content of the sheet product.

Table 4 shows the OES chemical analysis of the DPF samples. Again, no significant variation from the virgin powder’s composition was measured. The results show how the Ti-6Al-4V_DPF_ bars respect the ASTM B381-13 standard specification for titanium and titanium alloy forgings grade F-23 [15].

### 3.2. Microstructure Characterization and Phase Identification

**Figure 5:**
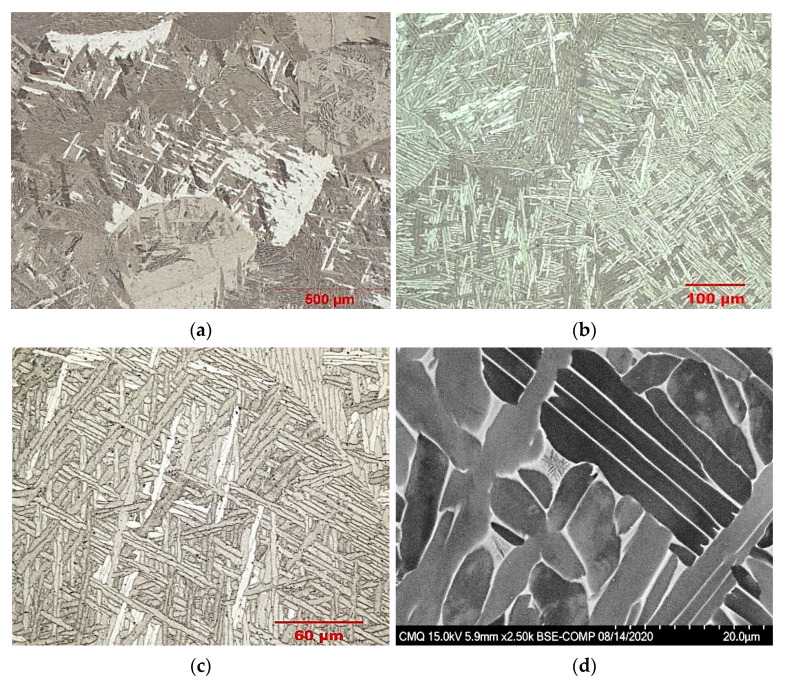
Ti-6Al-4V_DPF_ optical micrographs after annealing and furnace cooled. Representation of the average grain size (**a**). Higher magnification showing the lamellar microstructure (**b**,**c**). Backscattered electron micrograph showing the alpha lamella morphology (**d**).

The microstructure of the annealed Ti-6Al-4V_DPF_ sample is presented in Figure 5. Grain growth occurs as a result of the total deformation generated by the DPF process in conjunction with the annealing treatment, which is to be expected after continuous exposure at high temperature, i.e., 925 °C for 2 h [16]. Furthermore, the final grain size of Ti-6Al-4V_DPF_ is in agreement with the one reported by other researchers for wrought Ti-6Al-4V produced with similar annealing treatments [17]. Moreover, all samples presented a typical lamellar microstructure. The lamellae appear in colonies and exhibit different orientations. This behavior is caused by the multiple possibilities of reticular movement that occur during the allotropic transformation from the body-centered cubic (BCC) lattice to the hexagonal close-packed (HCP) lattice [18]. The lamellar microstructure also shows some strong similarities with the results reported by Weiss et al. for Ti-6Al-4V after β-phase thermomechanical processing and subsequent α+β annealing [19]. It was found that the thickness and length of the deformed and annealed alpha lamella is related to the amount of total strain applied during forging and the annealing parameters. The close relationship between the α-phase morphology and the amount of deformation has also been reported by other researchers [20], which shows good agreement with the microstructure of Ti-6Al-4V after DPF process. Therefore, the total deformation needed to produce a fully dense bar of Ti-6Al-4V_DPF_ combined with the annealing in the upper α+β region caused the breakup of the lamella into smaller sections and their thickening. For the latter, the holding at high temperature and the slow cooling from α+β annealing results in the increase in the size of α lamella as well as the β grain [17,21]. Additionally, no defect or residual porosity was observed by the optical analysis.

**Figure 6:**
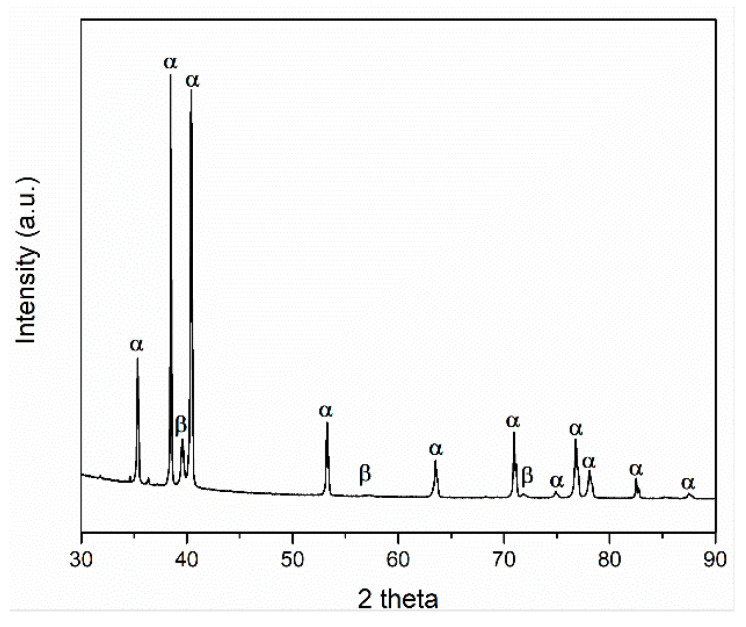
XRD pattern of Ti-6Al-4V_DPF_ after annealing at 925 °C for 2 h.

Figure 6 presents the Ti-6Al-4V_DPF_ XRD patterns after the annealing heat treatment. The identified phases confirm the α+β structure. The high intensity of the α phase found for the Ti-6Al-4V_DPF_ is likely explained by the crystallographic texture caused by the unidirectional hot rolling. This also indicates that the annealing at 925 °C did not eliminate the anisotropy of the microstructure.

Furthermore, image analysis was performed on the backscattered images providing an estimate of the proportion of α-phase at 88% and β-phase 12%. Table 5 presents the Rietveld refinement result of the XRD patterns presented in Figure 6. The α-phase fraction obtained from the Rietveld refinement was 92 wt-% this agrees with the phase fraction estimated from image analysis.

### 3.3. Canister-Alloy Interface Characterization

**Figure 7:**
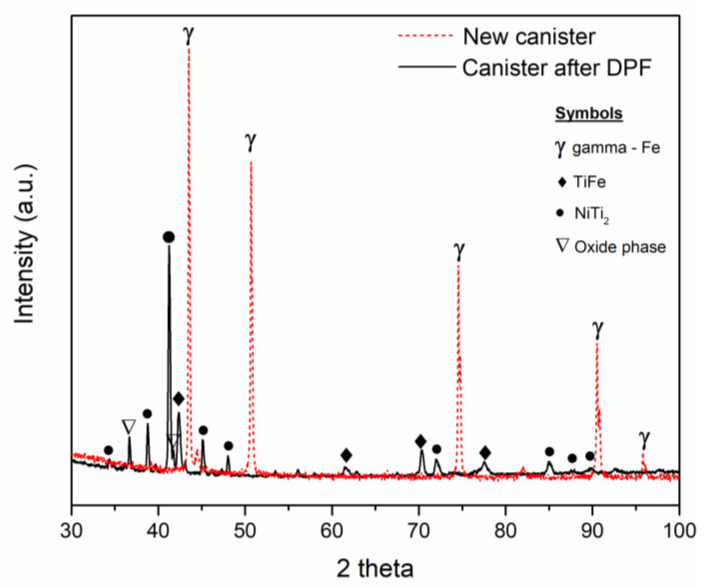
XRD pattern of the stainless steel canister inner wall before and after DPF.

To evaluate the reaction at the canister–alloy interface, XRD analysis was performed on the stainless-steel canister’s inner surface before and after DPF (see Figure 7). The surface of the new canister presented a well-defined gamma phase spectrum, typical of austenitic stainless steel. On the other hand, after DPF, the canister’s inner surface showed a completely different diffraction pattern, having defined peaks for TiFe, NiTi_2_ and complex oxides. These phases have been formed by diffusion at the interface during the sintering and the thermomechanical steps. This observation was also reported by Scherillo et al., who obtained a similar XRD pattern on stainless steel canisters of HIPped titanium specimens [22]. Nevertheless, as seen in Figure 6, none of these phases were identified in the bulk of the Ti-6Al-4V_DPF_ alloy.

**Figure 8:**
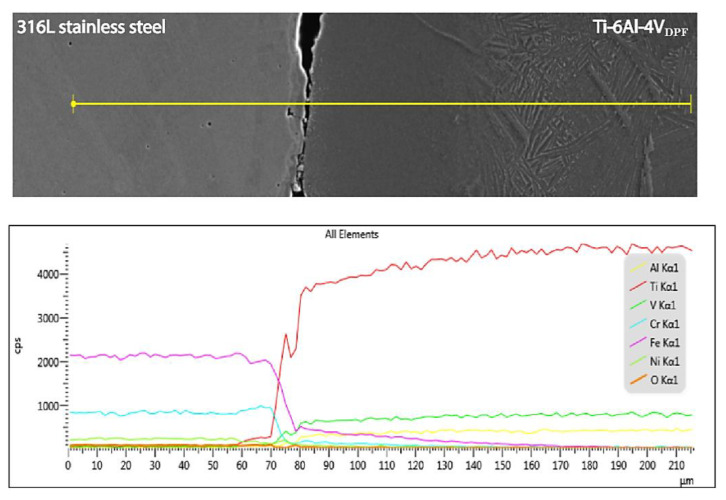
Typical backscatter electron micrograph and EDS linescan analysis of the interface between the stainless steel canister and the Ti-6Al-4V_DPF_.

Further characterization was performed by SEM/EDS to evaluate the chemical variation between the Ti-6Al-4V_DPF_ and the stainless steel canisters. Figure 8 presents an EDS linescan analysis that shows similar diffusion behavior for iron (Fe), chromium (Cr) and nickel (Ni) within the titanium alloy. We can also notice that the chemical composition returns to the nominal alloy’s values within 100 µm of the interface. This behavior is also mentioned by Scherillo et al. [22] and is expected when Ti-6Al-4V parts are HIPed with a stainless steel container. This result confirms that the surface contamination is limited with the DPF process.

As can be observed in Figure 8, by the final stages of the process, all DPF specimens do not present a continuous interface between the canisters and the alloy. The forging and rolling stages applied shear strain and stresses at the interface that fractured the intermetallic/oxides compounds that formed during DPF. De-feng et al. provided a detailed review on the diffusion between the two alloys and related this fracture phenomenon to their important disparity in physical properties and the brittle nature of the formed compounds [23]. Therefore, the reaction itself acts has an efficient stripping aid for the DPF samples. In this study, the limited bonding between the canister and the specimen allowed for a simple slide out extraction by cutting and manually removing the canister on all samples. The simplicity of extraction for Ti-6Al-4V_DPF_ can benefit industrial applications such as for the near-net-shape approach, which offers a new perspective for future investigations that require low interstitial components. As previously mentioned, research reported the potential of DPF for near-net-shape components, but there are still developments that need to be made [6].

### 3.4. Mechanical Properties

**Figure 9:**
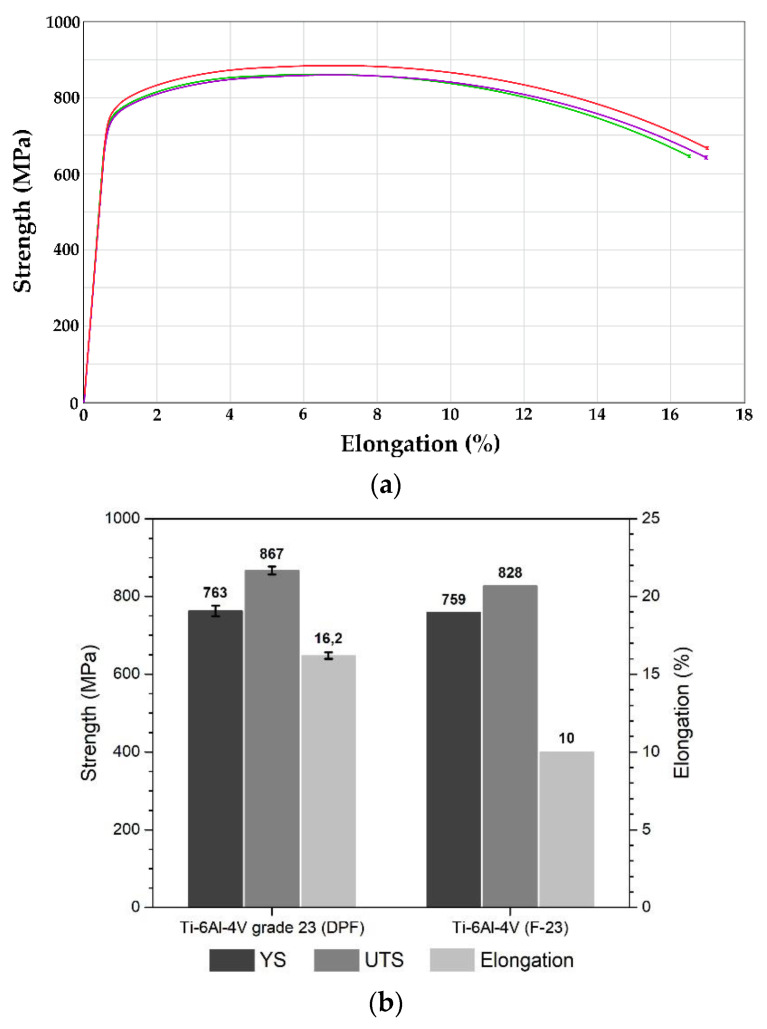
Mechanical properties of annealed Ti-6Al-4V_DPF_. Representative stress-strain curves are presented in (**a**) and the averages for YS, UTS and elongation compared to the standard requirement (grade F23) are presented in (**b**).

Figure 9 presents the averaged tensile mechanical properties of the bars produced by DPF. The yield strength (YS) and the ultimate tensile strength (UTS) of the Ti-6Al-4V_DPF_ show similar results over the ELI grade F-23 (ASTM standard B381-13). In contrast, elongation (16.2%) has been enhanced significantly when compared to standard requirements. The effect of the high temperature and the slow cooling parameters of the annealing treatment have been previously reported to show a significant decrease in elongation [17]. However, the Ti-6Al-4V_DPF_ obtained almost twice the elongation of the requirement while keeping similar tensile strength when compared to results reported by previous authors for wrought Ti-6Al-4V produced with similar heat treatments. Both α-phase morphology and volume fraction have a significant effect on the mechanical properties of Ti-6Al-4V. Similar lamellar microstructures as the ones observed on the Ti-6Al-4V_DPF_ have shown to be responsible for the decrease in the resistance to dislocation slip in the α phase, resulting in the increase in the plastic strain [24]. In addition, the elongation of Ti-6Al-4V is dramatically reduced when the oxygen concentration increases [25]. Since oxygen is an interstitial element, the rise in oxygen content impedes and disrupts twinning deformation, which can alter the dislocation mobility and enhance slip resistance [26,27]. From this standpoint, the higher ductility of the Ti-6Al-4V_DPF_ alloy when compared to ELI grade requirements can also be attributed to the low oxygen content (0.05 vs. 0.13 wt-%). As mentioned above, the noteworthy properties obtained for the Ti-6Al-4V_DPF_ can fulfill the need for ELI grade in near-net-shape components, which have shown superior tensile strength and elongation compared to the standard specification for Ti-6Al-4V PM LI [28]. Future works are needed to develop the full potential of DPF titanium alloy for the near-net-shape approach.

## 4. Conclusions

The goal of this study was to evaluate the feasibility of direct powder forging (DPF) as a technique to produce bulk titanium alloys from oversized spherical prealloyed titanium powder and to evaluate their mechanical properties in the annealed state. Ti-6Al-4V was used for the characterization of the process. The main findings of this research can be summarized as follows:

1. Full densification and homogeneous lamellar α+β microstructure were obtained for Ti-6Al-4V_DPF_. Wrought products can be produced from large spherical prealloyed titanium powder.
2. The chemical composition variation from the starting condition was negligible. High purity ELI grade was preserved for the Ti-6Al-4V_DPF_ sample.
3. EDS linescans and XRD analysis have shown that DPF causes comparable surface contamination to HIP processing of titanium alloys.
4. DPF causes high strain and stresses at the canister and alloy interface, which fractures the formed intermetallics/oxides and allows for an easy canister removal.
5. DPF process has produced a significant enhancement in ductility over the standard requirements for Ti-6Al-4V (F-23).

Future work will assess the possibility of producing a near-net-shape part with a final close-die forging step.

## Figures and Tables

**Table 1:** Chemical composition and average size of the base powder used.

	Chemical Composition (%-wt)								Size
	Ti	Fe	Al	V	O	S	C	Mn	D_50_ (µm)
Ti-6Al-4V	Bal.	0.16	6.43	4.15	0.04	---	<0.01	---	150

**Table 2:** Average titanium samples density before and after DPF.

		Density (g/cm^3^)		Relative Density (%)	
	Theoretical	Before DPF	After DPF	Before DPF	After DPF
Ti-6Al-4V	4.430	2.787 ± 0.010	4.420 ± 0.004	62.9	99.8

**Table 3:** Oxygen analysis of the original powder and of the DPF samples.

	Oxygen (wt-%)
* Ti-6Al-4V	0.04
Ti-6Al-4V_DPF_	0.050 ± 0.002

* Chemistry from the supplier.

**Table 4:** OES chemical analysis of the produced alloys (wt-%).

	Fe	Ti	Al	V	C
* Ti-6Al-4V	0.16	Bal.	6.43	4.15	<0.01
Ti-6Al-4V_DPF_	0.16	Bal.	6.29	4.09	<0.01

* Chemistry from the supplier.

**Table 5:** Rietveld refinement of Ti-6Al-4V_DPF_ after annealing at 925 °C for 2 h.

	Phase	Phase Fraction (wt-%)	Lattice Parameters (Å)
Ti-6Al-4VDPF	Ti-alpha (α)	92	a = 2.93 (7) ^†^
			c = 4.67 (1)
	Ti-beta (β)	8	a = 3.21 (3)

^†^ The uncertainty on the last significant digit is given by the number in parenthesis.
